# Mortality and Incidence of Hospital Admissions for Stroke among Brazilians Aged 15 to 49 Years between 2008 and 2012

**DOI:** 10.1371/journal.pone.0152739

**Published:** 2016-06-22

**Authors:** Fernando Adami, Francisco Winter dos Santos Figueiredo, Laércio da Silva Paiva, Thiago Hérick de Sá, Edige Felipe de Sousa Santos, Bruno Luis Martins, Vitor Engrácia Valenti, Luiz Carlos de Abreu

**Affiliations:** 1 Departamento de Saúde da Coletividade, Laboratório de Epidemiologia e Análise de Dados, Faculdade de Medicina do ABC, Santo André, São Paulo, Brasil; 2 Departamento de Saúde da Coletividade, Laboratório de Delineamento de Estudos e Escrita Científica, Faculdade de Medicina do ABC, Santo André, São Paulo, Brasil; 3 Departamento de Epidemiologia, Faculdade de Saúde Pública da Universidade de São Paulo, São Paulo, São Paulo, Brasil; 4 Departamento de Nutrição da Faculdade de Saúde Pública, Universidade de São Paulo, São Paulo, São Paulo, Brasil; 5 Unidade Saúde, Faculdade Leão Sampaio, Juazeiro do Norte, Ceará, Brasil; 6 Departamento de Fonoaudiologia, Faculdade de Filosofia e Ciências da Universidade Estadual Paulista Júlio de Mesquita Filho, Marília, São Paulo, Brasil; BRAC, BANGLADESH

## Abstract

**Introduction:**

The objective was to analyze rates of stroke-related mortality and incidence of hospital admissions in Brazilians aged 15 to 49 years according to region and age group between 2008 and 2012.

**Methods:**

Secondary analysis was performed in 2014 using data from the Hospital and Mortality Information Systems and the Brazilian Institute of Geography and Statistics. Stroke was defined by ICD, 10th revision (I60–I64). Crude and standardized mortality (WHO reference) and incidence of hospital admissions per 100,000 inhabitants, stratified by region and age group, were estimated. Absolute and relative frequencies; and linear regression were also used. The software used was Stata 11.0.

**Results:**

There were 35,005 deaths and 131,344 hospital admissions for stroke in Brazilians aged 15–49 years old between 2008 and 2012. Mortality decreased from 7.54 (95% CI 7.53; 7.54) in 2008 to 6.32 (95% CI 6.31; 6.32) in 2012 (β = -0.27, p = 0.013, r^2^ = 0.90). During the same time, incidence of hospital admissions stabilized: 24.67 (95% CI 24.66; 24.67) in 2008 and 25.11 (95% CI 25.10; 25.11) in 2012 (β = 0.09, p = 0.692, r^2^ = 0.05). There was a reduction in mortality in all Brazilian regions and in the age group between 30 and 49 years. Incidence of hospitalizations decreased in the South, but no significant decrease was observed in any age group.

**Conclusion:**

We observed a decrease in stroke-related mortality, particularly in individuals over 30 years old, and stability of the incidence of hospitalizations; and also regional variation in stroke-related hospital admission incidence and mortality among Brazilian young adults.

## Introduction

Stroke is the second most common cause of death in the world [1] and the first in Brazil. [2] In low- and middle-income countries, mortality from stroke is reducing, while incidence is increasing in the last years. [3] Brazil is an upper middle-income country with 206.1 million inhabitants (http://data.worldbank.org/country/brazil) that suffers from serious problems of social inequality and triple burden of disease (infectious, non-communicable and external causes). [4]

There is a lack of studies estimating stroke mortality and incidence in younger populations, including in Brazil. Updated and representative data are necessary to estimate stroke burden in the country as most studies on stroke conducted in Brazil were published before 2002,[5] included all age groups or had very specific study population, [6, 7] limiting the generalizability of the estimates. Therefore, it is difficult to identify whether there is a reduction in mortality and incidence of stroke in the younger subgroup of the population, especially given the increasing prevalence of risk factors, such as diabetes mellitus, hypertension and dyslipidemia [8] among young individuals worldwide. [9] In Brazil, there are also few studies on risk factors for stroke in younger populations. [10]

To address the gap regarding estimates on the burden of stroke in Brazilian young population, this study aimed to analyze stroke-related mortality and incidence of hospital admissions in Brazilians aged 15 to 49 years between 2008 and 2012, by using data provided by the Department of Informatics of the National Health System (DATASUS), which include 96.1% of deaths reports in the country [11] and is maintained by the Brazilian Ministry of Health.

## Methods

This study was performed in October 2014 by a secondary analysis of mortality and hospitalization data from residents of Brazil (thereafter, Brazilians) collected, respectively, from the Mortality Information System (SIM) and the Hospital Information System (SIH). Both systems are included in the Brazilian Health Ministry database, available at the DATASUS website (www.datasus.gov.br). The study was approved by the Ethics Committee on Human Research of the Faculty of Medicine of ABC (process number 214586).

### Information Systems

The SIM and the SIH receive, process, check the validity and make the information available of deaths (SIM) and hospitalizations (SIH) of people seeking one of the registered health facilities in the The National Health System (Sistema Único de Saúde—SUS), which comprises 92.3% [12] of health facilities in Brazil. Data on deaths and hospitalizations are thus available on the website of the Department of Informatics of the The National Health System (DATASUS), which constitutes the official, free and public database of health information in the country, from which we collected the information on death and hospitalization for stroke used in this study. It is noteworthy that such systems are used for the development of public policies in the country. Population size was obtained from the last census of the Brazilian population performed in 2010 and from projections for the other years. The estimates were performed by the Brazilian Institute of Geography and Statistics (IBGE—www.ibge.gov.br), also available in DATASUS webpage.

Data on deaths and hospitalizations for stroke were collected using the tenth revision of the International Classification of Diseases (I60–I64 codes) [13] for the total population and stratified according to age groups (15–19; 20–29; 30–39; 40–49 years), regions (North, Northeast, Southeast, South and Central-west) and calendar years (2008, 2009, 2010, 2011 and 2012), as available in DATASUS database. All these data were extracted and unified in TabNet program, creating a database of DBF type.

### Variables and Data Extraction

We calculated mortality and incidence of hospital admission for stroke, expressed as rate per 100,000 inhabitants. Crude and age-standardized estimates, having WHO world population as the reference [14], were calculated. Mortality-to-incidence ratio was also estimated by the ratio between number of deaths and hospital admission according to sex, age group, country regions, calendar year and stroke subtypes, aimed at assessing success or failure of health services in stroke management [3]. Proportional mortality was estimated by ratio between number of deaths by stroke (I60-I64) and number of death by all causes (excluding R00 to R99).

To assess the quality of the information systems, we estimated the proportion of deaths from ill-defined causes (ICD 10 codes R00 to R99) [13] for each year. All compilation of DATASUS data was performed by two researchers independently using extraction sheets designed by the authors; a third investigator was responsible for correcting discrepancies.

### Statistical analysis

To describe stroke-related mortality and incidence, we used absolute and relative frequencies; and also 95% confidence intervals (95% CI). To estimate trend of stroke-related mortality and incidence, we used linear regression, estimating the slope (β), its respective probability (p), and the model's predictive ability (r^2^): The regression model used [15] is described below:

y = β0+β1*x, being

y = Mortality or incidence of hospitalizations for stroke in Brazilians aged 15 to 49 years (per 100,000 Brazilians aged 15 to 49 years);

x = calendar years (2008, 2009, 2010, 2011 e 2012);

β0 = linear coefficient;

β1 = angular coefficient, which represents the mean annual variation of deaths or hospitalizations for stroke in Brazilians aged 15 to 49 years old. For example, a β1 equal to -1 means that, for each year, there was a reduction of one death or one hospitalization for stroke per 100,000 Brazilians aged 15 to 49 years. In this model, r^2^ was also obtained, which represents the explanatory capacity of the linear model used to assess the mortality as a function of the years during the study period. The significance level was 5%. The statistical program was Stata 11.0.

## Results

**Fig 1:**
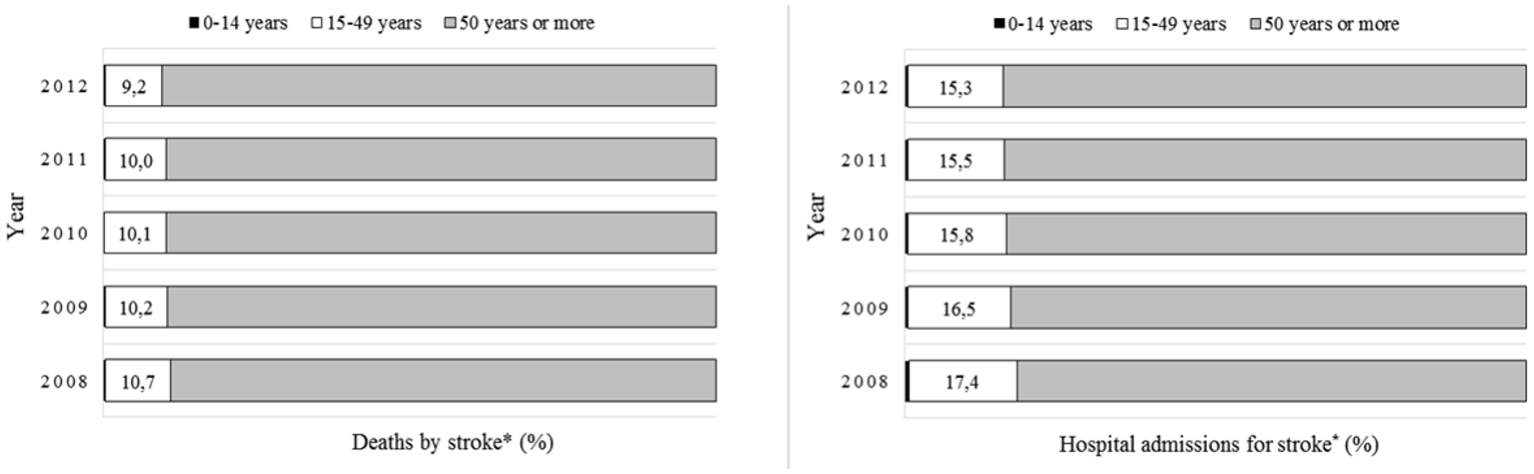
Distribution of deaths and hospital admissions for stroke* in Brazilians between 2008 and 2012. * International classification of diseases, 10th revision. Codes I60 to I64. [11] Source: Mortality Information System (SIM) and Hospital Information System (SIH/SUS). Data made available by the Department of Informatics of The National Health System (DATASUS—www.datasus.gov.br). Ministry of Health, Brazil.

We found 35,005 deaths and 131,344 hospital admissions caused by stroke in Brazilians aged 15–49 years during 2008–2012 (Table 1), representing on average 10.0% (range during this period: 10.7% to 9.2%) and 16.1% (range during this period: 17.4% to 15.3%) of all deaths and hospital admissions for stroke in Brazil (Fig 1).

**Table 1:** Burden of stroke^†^ in Brazilians aged 15 to 49 years during 2008–2012.

Characteristics	Deaths	Age- standardized mortality (95% CI)	Hospital admissions	Age-standardized Incidence (95% CI)	Mortality-to-incidence (95% CI)	Proportional mortality (95% CI)
Sex						
Male	17,696	7.12 (7.11 to 7.12)	65,673	26.17 (26.16 to 26.17)	0.27 (0.26 to 0.27)	2.25 (2.24 to 2.25)
Female	17,308	6.56 (6.55 to 6.56)	65,671	24.80 (24.79 to 24.80)	0.26 (0.25 to 0.26)	5.72 (5.69 to 5.74)
Age group (years)*						
15–19	675	0.79 (0.78 to 0.79)	4,702	5.52 (5.51 to 5.52)	0.14 (0.13 to 0.14)	0.72 (0.71 to 0.72)
20–29	2,629	1.51 (1.50 to 1.51)	15,349	8.81 (8.80 to 8.81)	0.17 (0.16 to 0.17)	0.98 (0.97 to 0.98)
30–39	7,745	5.26 (5.25 to 5.26)	31,823	21.61 (21.60 to 21.61)	0.24 (0.23 to 0.24)	2.66 (2.65 to 2.66)
40–49	23,956	19.45 (19.44 to 19.45)	79,470	64.43 (64.40 to 64.45)	0.30 (0.29 to 0.30)	5.52 (5.50 to 5.53)
Country regions						
North	2,502	6.89 (6.88 to 6.89)	7,854	20.94 (20.92 to 20.95)	0.33 (0.32 to 0.33)	2.98 (2.95 to 3.00)
Northeast	9,199	7.03 (7.02 to 7.03)	31,840	23.76 (23.75 to 23.76)	0.30 (0.29 to 0.30)	2.93 (2.91 to 2.94)
Southeast	16,394	7.20 (7.19 to 7.20)	58,049	25.53 (25.52 to 25.53)	0.28 (0.27 to 0.28)	3.67 (3.65 to 3.68)
South	4,510	5.72 (5.71 to 5.72)	23,840	30.53 (30.51 to 30.54)	0.19 (0.18 to 0.19)	2.89 (2.87 to 2.90)
Central-west	2,400	6.21 (6.20 to 6.21)	9,761	24.99 (24.97 to 25.00)	0.25 (0.24 to 0.25)	2.75 (2.73 to 2.76)
Stroke subtypes^†^						
Ischemic (I63)	1,296	0.25 (0.24 to 0.25)	10,233	1.99 (1.98 to 1.99)	0.13 (0.12 to 0.13)	0.12 (0.11 to 0.12)
Hemorrhagic (I60-I62)	22,146	4.32 (4.31 to 4.32)	50,171	9.71 (9.70 to 9.71)	0.44 (0.43 to 0.44)	2.04 (2.03 to 2.04)
Nonspecified** (I64)	11,563	2.27 (2.26 to 2.27)	70,940	13.78 (13.77 to 13.78)	0.16 (0.15 to 0.16)	1.06 (1.05 to 1.06)

* crude rate

** Stroke not specified as ischemic or hemorrhagic

^†^ International classification of diseases. 10th revision. Codes I60 to I64. [11]

Source: Mortality Information System (SIM) and Hospital Information System (SIH/SUS). Data made available by the Department of Informatics of The National Health System (DATASUS—www.datasus.gov.br). Ministry of Health. Brazil.

Of all deaths from stroke in individuals aged 15–49 years during 2008–2012, 17,696 occurred in men and 17,308 in women, representing 2.25% (95% CI 2.24; 2.25%) and 5.72% (95% CI 5.69%; 5.74%) of all deaths by defined causes, respectively. There is one individual for which sex was ignored. The number of hospital admissions for stroke did not differ between men (n = 65,673) and women (n = 65,671), but age-standardized incidence of hospital admissions per 100,000 inhabitants was higher in men (26.17, 26.16 to 26.17) than in women (24.80, 24.79 to 24.80). All indicators are increasing with age group, with greater impact from the age of 30 (Table 1).

The largest number of deaths and hospital admissions and proportional mortality occurred in the Southeast (n = 16,394, n = 58,049 and 3.67%, respectively). Higher age-standardized mortality rate, age-standardized incidence of hospital admission and mortality-to-incidence ratio was found in the Southeast with 7.20 (95% CI 7.19 to 7.20), South with 30.53 (95% CI 30.51 to 30.54) and North with 0.33 (95% CI 0.32 to 0.33), respectively (Table 1).

The most common cause of death and proportional mortality was hemorrhagic stroke (ICD I60 and I62) (n = 22,146 and 2.04%, 95% CI 2.03 to 2.04), followed by unspecified stroke (ICD I64) (n = 11,563 and 1.06, 95% CI 1.05 to 1.06) (Table 1).

The age-standardized mortality from stroke in Brazil decreased from 7.54 (95% CI 7.53–7.54) to 6.32 (95% CI 6.31–6.32) per 100,000 inhabitants from 2008 to 2012 (β = -0.27, p = 0.013, r^2^ = 0.90), a decrease of 16% (95% CI 16; 17). There was a reduction in mortality in all regions, with the highest predictive ability in the North (β = -0.38, p = 0.008, r^2^ = 0.93) and lowest in the Central-west (β = -0.31, p = 0.077, r^2^ = 0.70). During the years 2008 to 2012, the lowest mortality from stroke (per 100,000 inhabitants) was in the South, ranging from 6.32 (95% CI 6.31; 6.32) to 5.10 (95% CI 5.09; 5.10); the largest was in the Southeast, ranging from 8.07 (95% CI 8.06; 8.07) to 6.65 (95% CI 6.64; 6.65) (Table 2).

**Table 2:** Mortality* and incidence* of hospital admissions for stroke^†^ (95% confidence interval) per 100,000 inhabitants; and estimates of linear regression (referring to the rates) in Brazilians aged 15 to 49 years between 2008 and 2012, according to country regions and year.

Brazil/Region	Age-standardized Mortality* (95% CI) by stroke† (x100,000 inhabitants)					Linear Regression		
	2008	2009	2010	2011	2012	β	*p*	r^2^
Brazil	7.54 (7.53; 7.54)	6.93 (6.92; 6.93)	6.73 (6.72; 6.73)	6.66 (6.65; 6.66)	6.32 (6.31; 6.32)	-0.27	0.013	0.90
North	7.69 (7.68; 7.69)	7.40 (7.39; 7.40)	6.73 (6.72; 6.73)	6.31 (6.30; 6.31)	6.30 (6.29; 6.30)	-0.38	0.008	0.93
Northeast	7.40 (7.39; 7.40)	7.30 (7.29; 7.30)	6.83 (6.82; 6.83)	6.94 (6.93; 6.94)	6.66 (6.65; 6.66)	-0.18	0.024	0.85
Southeast	8.07 (8.06; 8.07)	7.19 (7.18; 7.19)	7.07 (7.06; 7.07)	7.04 (7.03; 7.04)	6.65 (6.64; 6.65)	-0.29	0.037	0.81
South	6.32 (6.31; 6.32)	5.66 (5.65; 5.66)	6.03 (6.02; 6.03)	5.51 (5.50; 5.51)	5.10 (5.09; 5.10)	-0.25	0.056	0.75
Central-west	7.21 (7.20; 7.21)	6.24 (6.23; 6.24)	5.82 (5.81; 5.82)	6.10 (6.09; 6.10)	5.70 (5.69; 6.70)	-0.31	0.077	0.70
Brazil/Region	Age-standardized Incidence* (95% CI) of hospital admissions for stroke^†^ (x100,000 inhabitants)					Linear Regression		
	2008	2009	2010	2011	2012	β	*p*	r^2^
Brazil	24.67 (24.66; 24.67)	25.99 (25.98; 25.99)	25.63 (25.62; 25.63)	26.01 (26.00; 26.01)	25.11 (25.10; 25.11)	0.09	0.692	0.05
North	17.64 (17.62; 17.65)	23.23 (23.21; 23.24)	21.71 (21.69; 21.72)	20.59 (20.57; 20.60)	21.54 (21.52; 21.55)	0.51	0.512	0.15
Northeast	20.66 (20.65; 20.66)	23.53 (23.52; 23.53)	23.18 (23.17; 23.18)	26.52 (26.51; 26.52)	24.90 (24.89; 24.90)	1.14	0.079	0.69
Southeast	25.46 (25.45; 25.46)	25.99 (25.98; 25.99)	25.93 (25.92; 25.93)	25.56 (25.55; 25.56)	24.72 (24.71; 24.72)	-0.19	0.291	0.35
South	32.10 (32.08; 32.11)	31.33 (31.31; 31.34)	30.89 (30.87; 30.90)	29.22 (29.20; 29.23)	29.13 (29.11; 29.14)	-0.80	0.007	0.93
Central-west	24.66 (24.64; 24.67)	25.91 (25.89; 25.92)	25.34 (25.32; 25.35)	25.52 (25.50; 25.53)	23.53 (23.51; 23.54)	-0.26	0.449	0.20

* Standardized for age according to the world population from the World Health Organization. [10]

^†^ International classification of diseases, 10th revision. Codes I60 to I64. [11]

β—regression slope; r^2^—predictive capacity; 95% CI—95% confidence interval. Source: Mortality Information System (SIM) and Hospital Information System (SIH/SUS). Data made available by the Department of Informatics of The National Health System (DATASUS—www.datasus.gov.br). Ministry of Health, Brazil.

In Brazil, the incidence of hospital admissions for stroke (per 100,000 inhabitants) remained stable: 24.67 (95% CI 24.66; 24.67) in 2008 and 25.11 (95% CI 25.10; 25.11) in 2012 (β = 0.09, p = 0.692, r^2^ = 0.05). The South region had the highest incidence, despite decreasing estimates, ranging from 32.10 (95% CI 32.08; 32.11) in 2008 to 29.13 (95% CI 29.11; 29.14) in 2012 (β = -0.80, p = 0.007, r^2^ = 0.93). There was an increase in the Northeast region, ranging from 20.66 (95% CI 20.65; 20.66) to 24.90 (95% CI 24.89; 24.90) (β = 1.14, p = 0.079, r^2^ = 0.69). The North region had the lowest incidence of hospital admissions, with no decrease in rates (Table 2).

Between 2008 and 2012, there was a decrease in age-standardized mortality from stroke (per 100,000 inhabitants) in the age groups 30 to 39 years, from 5.73 (95% CI 5.72; 5.73) to 4.92 (95% CI 4.91; 4.92) (β = -0.17, p = 0.020, r^2^ = 0.87), and 40 to 49 years, from 21.75 (95% CI 21.74; 21.75) to 17.80 (95% CI 17.79; 17.80) (β = -0.87, p = 0.018, r^2^ = 0.88) (Table 3).

**Table 3:** Mortality* and incidence* of hospital admissions for stroke^†^ (95% confidence interval) per 100,000 inhabitants; and estimates of linear regression (referring to the rates) in Brazilians from 15 to 49 years between 2008 and 2012, by age group and year.

Age group (years)	Mortality* (CI 95%) by stroke^†^ (x100,000 inhabitants)					Linear Regression		
	2008	2009	2010	2011	2012	β	*p*	r^2^
15–19	0.81 (0.80; 0.81)	0.83 (0.82; 0.83)	0.73 (0.72; 0.73)	0.76 (0.75; 0.76)	0.83 (0.82; 0.83)	-0.003	0.866	0.01
20–29	1.53 (1.52; 1.53)	1.52 (1.51; 1.52)	1.58 (1.57; 1.58)	1.46 (1.45; 1.46)	1.45 (1.44; 1.45)	-0.02	0.236	0.42
30–39	5.73 (5.72; 5.73)	5.27 (5.26; 5.27)	5.27 (5.26; 5.27)	5.14 (5.13; 5.14)	4.92 (4.91; 4.92)	-0.17	0.020	0.87
40–49	21.75 (21.74; 21.75)	19.79 (19.78; 19.79)	18.95 (18.94; 18.95)	18.97 (18.96; 18.97)	17.80 (17.79; 17.80)	-0.87	0.018	0.88
Age group (years)	Incidence* of hospital admissions (CI 95%) by stroke^†^ (x100,000 inhabitants)					Linear Regression		
	2008	2009	2010	2011	2012	β	*p*	r^2^
15–19	5.59 (5.58; 5.59)	5.40 (5.39; 5.40)	5.62 (5.61; 5.62)	5.54 (5.53; 5.54)	5.47 (5.46; 5.47)	-0.01	0.777	0.03
20–29	8.57 (8.56; 8.57)	8.61 (8.60; 8.61)	9.23 (9.22; 9.23)	9.03 (9.02; 9.03)	8.62 (8.61; 8.62)	0.05	0.655	0.07
30–39	21.19 (21.18; 21.19)	21.81 (21.80; 21.81)	21.74 (21.73; 21.74)	21.77 (21.76; 21.77)	21.53 (21.52; 21.53)	0.06	0.513	0.15
40–49	61.82 (61.79; 61.84)	66.61 (66.58; 66.63)	64.34 (64.31; 64.36)	66.11 (66.08; 66.13)	63.28 (63.25; 63.30)	0.24	0.756	0.03

* Crude rate

^**†**^ International classification of diseases, 10th revision. Codes I60 to I64. [11]

β—regression slope; r^2^—predictive capacity; 95% CI—95% confidence interval. Source: Mortality Information System (SIM) and Hospital Information System (SIH/SUS). Data made available by the Department of Informatics of The National Health System (DATASUS—www.datasus.gov.br). Ministry of Health, Brazil.

There was no variation in the incidence of hospital admissions for stroke (per 100,000 inhabitants) according to age groups; in 2012, the incidence was 5.47 (95% CI 5.46; 5.47) for people aged 15 to 19 years and 63.28 (95% CI 63.25; 63.30) for those aged 40 to 49 years (Table 3).

**Fig 2:**
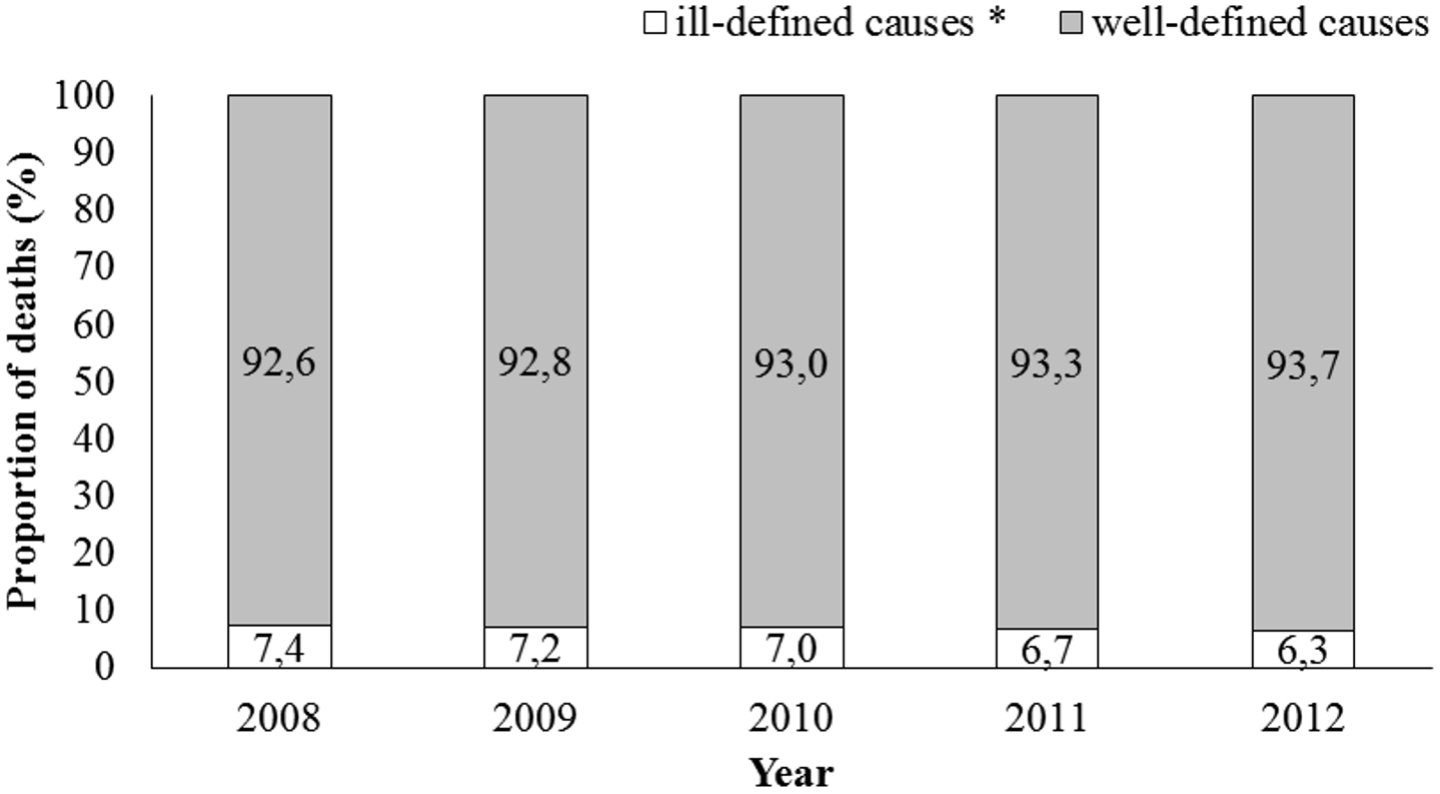
Proportion of deaths from ill-defined causes* of Brazilians between 2008 and 2012. * International classification of diseases, 10th revision. Codes R00 to R99. [11] Source: Mortality Information System (SIM) and Hospital Information System (SIH/SUS). Data made available by the Department of Informatics of The National Health System (DATASUS—www.datasus.gov.br). Ministry of Health, Brazil.

The proportion of deaths from ill-defined cause was below 7.5%, ranging from 7.4% in 2008 to 6.3% in 2012 (Fig 2).

## Discussion

This study represents one of the first to analyze the profile of deaths and hospital admissions for stroke in younger population of a developing, middle-income country, with approximately 200 million people. Data obtained from information systems maintained by the Brazilian Ministry of Health, already used in previous studies [16] [17], point to a 16% reduction in mortality from stroke (7.54 to 6.32 per 100,000 inhabitants) among young people (15–49 years) in the five country regions, and a stabilization, on average, in the incidence of hospital admissions for stroke (24.67 to 25.11 per 100,000 inhabitants), although there was a reduction in the South and an increase in the Northeast.

Brazil is divided into five regions. The Southeast has the greatest number of inhabitants, followed by South, Northeast, Central-west and North region [18]. Mortality from stroke was found to be decreasing in other populations, including other Brazilian subgroups, pointing to a scenario of decreasing risk of death from stroke in recent years. Feigin et al. [3] showed an average reduction of 37% (95% CI 31%; 41%) in mortality from stroke in high-income countries and 20% (95% CI 15%; 30%) in low- and middle-income countries between 1990 and 2010.

Garritano et al. [19] found a reduction of 15% in mortality from stroke between 2000 and 2009 in Brazilians over 30 years old. In Joinville, a city in southern Brazil, there was a reduction in risk of death from stroke by 37% between 1995 and 2006. [6] André et al. [5] described a downward trend in mortality from stroke in Brazil during three consecutive decades. This study shows that people under age 50 may already be part of this favorable scenario.

This reduction in mortality can be attributed to the decrease in stroke lethality, given the stability in the incidence of ischemic stroke and the reduction in the incidence of hemorrhagic stroke (which has greater lethality) in low- and middle-income countries, including Brazil. [20] Nevertheless, such country estimates should be viewed with caution, since there is a high proportion of admissions for unspecified stroke (54% between 2008 and 2012), which affects the risk estimates stratified by stroke subtypes, but not the estimates of this study, given that they were not stratified by stroke subtype.

Despite the reduction in mortality from stroke, the prevalence of risk factors for this outcome, such as overweight, physical inactivity and hypertension, is increasing, even in younger people. [2] Lackland et al. [21] point out that the decline in mortality from stroke is associated with improvement in health service conditions and in interventions based on scientific evidence, such as increasing public programs for prevention of risk factors for stroke in high-risk areas.

Data on incidence of hospital admissions for stroke or stroke itself in people younger than 50 years are scarce. Cabral et al. [6] showed significant reduction in the incidence of stroke from 29.6 to 20.5 (per 100,000 inhabitants) between 1995 and 2005/06 in the city of Joinville (southern Brazil). In accordance with these findings, we observed declining rates in the incidence of hospitalizations for stroke only in the South of Brazil. On the other hand, we found higher estimates: 29.13 (95% CI 29.11; 29.14) per 100,000 inhabitants. This difference can be explained by factors such as the study population (South region vs. Joinville in 2012) or the definition of the outcome (new case of stroke versus hospital admission for stroke).

These factors may also explain the difference between our findings and those of Cabral et al., [6] in the south of Brazil, who did not find any hospital admissions for people under 29 years old, in contrast to the 20,051 admissions found in our study in people under 30 years. The incidence of hospitalizations for stroke in people under 30 years old in Brazil between 2008 and 2012 is stable and is lower than in USA. [22] However, the importance of monitoring the deaths and hospitalizations for stroke in younger people should be emphasized, as well as the efforts needed to improve these rates in this age group. Data concerning the increase in risk factors for stroke in children and adolescents [2] further highlight the need of monitoring.

The only data that showed an increase (though moderate) in rates in the present study was the incidence of hospital admissions in the Northeast, which is likely to be related to socioeconomic level, since the region has approximately 61% of the 1,431 Brazilian municipalities with low human development index. [18]

The information systems used provide data on deaths and hospitalizations for stroke across the country, making it possible to estimate the national stroke burden and to analyse time trends. However, some limitations should be highlighted. The Hospital Information System (SIH/SUS) does not distinguish first admissions from readmissions, and therefore may overestimate the incidence of stroke because it does not exclude patients admitted for stroke in previous years or estimates on number of non-hospitalized stroke patients in Brazil. There is also the possibility of underestimating estimates due to incomplete coverage of deaths and hospital admissions and validity of information provided by the SIH/SUS, although coverage is high [11, 23], or due to the quality of record, although the proportion of deaths from ill-defined causes was close to 7% between 2008 and 2012.

Thus, in addition to studies on the occurrence of stroke in specific subgroups of the population, there is also need for studies monitoring trends in risk factors, such as tobacco, distribution of medications, quality of health care and socioeconomic conditions, because the consequences of increased prevalence of risk factors may, in the long run, change the scenario of mortality and incidence of stroke observed in this study.

## Conclusion

By analyzing stroke-related mortality and incidence in Brazilian young adults, we found evidence of decreased mortality, particularly in individuals over 30 years old, and stability of the incidence of hospitalizations; and also regional variation in both stroke-related mortality and hospital admission incidence among Brazilian young adults.

## Abbreviations

DATASUS: Department of Informatics of the National Health System
IBGE: Brazilian Institute of Geography and Statistics
SIM: Mortality Information System
SIH/SUS: Hospital Information System
ICD: International Classification of Diseases
WHO: World Health Organization
95% CI: 95% confidence interval
